# Editorial: Revisiting cellular metabolism and type 1 diabetes

**DOI:** 10.3389/fendo.2023.1175213

**Published:** 2023-05-04

**Authors:** Vikash Chandra, Latif Rachdi, Swetha Gopalakrishnan

**Affiliations:** ^1^Stem Cells and Metabolism Research Program, Faculty of Medicine, University of Helsinki, Helsinki, Finland; ^2^Institut Cochin, Université Paris Cité, L’Institut National de la Santé et de la Recherche Médicale (INSERM) U1016, Centre National de la Recherche Scientifique (CNRS) UMR 8104, Paris, France; ^3^Molecular and Integrative Biosciences Program (MIBS), Faculty of Biological and Environmental Sciences, University of Helsinki, Helsinki, Finland; ^4^Institute of Biotechnology, University of Helsinki, Helsinki, Finland

**Keywords:** type 1 diabetes, T1D autoimmunity, T1D mouse model, stem cell modeling, pancreatic beta (β) cells

Type 1 diabetes (T1D) is a chronic autoimmune condition characterized by islet inflammation resulting from poorly defined combinations of genetic, metabolic, immunologic, and environmental factors. The pathology elicits profound changes in energy metabolism and mitochondrial function in insulin-deprived conditions of the affected individuals. Several studies point to changes in systemic metabolites other than glucose levels in T1D patients. Reduced serum levels of succinic acid and phosphatidylcholine in neo-natal patients of T1D highlight the precedence of metabolic perturbation in the body before the full onset of β-cell autoimmunity. T1D patients are also reported to have differential fatty acid metabolism as compared to their healthy counterparts. Further, methionine deficiency in early childhood is proposed to be a possible risk factor to develop T1DM. Even though many studies highlight the changes in body metabolism in T1D conditions, we know very little about the cause and effect of changes in β-cell energy metabolism induced by risk factors a major one being the inflammatory milieu in TID. In the present Research Topic, we aimed to provide a collection of papers on the emerging concepts on modelling of T1D pathophysiology for example, the effect of pro-inflammatory cytokines on β-cells, how cellular communication with immune cells affect metabolic reprogramming in β-cells. In this topic call, a total five articles have been published of which two are original research articles, two reviews and one perspective.

In the first perspective article, Wong et al. compared 10 different machine learning algorithms which are currently used in big data analysis and identified set of genes associated with insulin transcription and validate it in the established T1D preclinical mouse model (Ire1αβ^-/-^) and T2D individuals big data sets. Interestingly, they found candidate genes including metabolic genes *APP, ADCYAP1, LDHA* and *SST* that were down regulated in the T1D preclinical mouse model (Ire1αβ^-/-^) suggesting a possible role of de-differentiation of β-cells in this model. It will be interesting to see if changes in metabolic genes is a cause or consequence to the proposed de-differentiation of β-cells. Additionally, this work also provides technical input to the β-cell community with publicly available codes/scripts for other researcher to use in existing as well as emerging datasets to identify genes or genetic pathways associated with diabetes.

The second article from Houeiss et al. is a comprehensive article that reviews our current understanding on different humanized HLA and autoantigen transgenic mice models and discussed how such models can be used to develop antigen specific immunotherapy for the restoration of immune tolerance in T1D patients. This strategy will aid in identifying new biomarkers and design new screening assays for early detection of the disease. Moreover, these personalized *in vivo* models will be an ideal tool to test the cross-talk between differentiated human stem cells derived islets (SC-islets) and hemopoietic stem progenitors of the same patient.

ER stress has long been considering as a major contributor for the β-cell failure in T1D and T2D. In the third original research article by Vig et al., highlights the previously unrecognized role of mitochondrial DNA (mtDNA) leakage in the cytosol of stressed β-cell which triggers IFN-I responses and consequently stimulates IL8 secretion resulting in neutrophil migration. They have employed a valid human β-cell line model EndoC-βh1 to demonstrate this phenomenon. This observation further strengths the involvement of β-cell ER stress in activating the innate immune responses which triggers immune-cells infiltration and sparked the autoimmunity.

The antigen-specific immunotherapy that can restore the immune tolerance to disease-relevant antigen has been long sought for the treatment of autoimmune diseases however there are several limitations to this therapy like timing, dose, route of administration etc. In the fourth original research article Martens et al. investigated the insulin peptide antigen-specific immunotherapy for T1D and shown how the addition of adjuvants like alum enhanced the efficacy of insulin B:8-24 peptide treatment in NOD mice model. Furthermore, they showed that inclusion of alum as adjuvant enhanced the tolerogenic response to the antigen accompanied with increased frequencies of insulin-reactive FoxP3^+^ Tregs and reduced frequencies of activated pathogenic CD4^+^ and CD8^+^ T cells in the pancreas of treated mice.

The purpose of the 5^th^ review paper was to summarize the progress and challenges in studying the *in vitro* β-cell killing models using immune cells and SC-islets (Halliez et al.). Here, authors elegantly compared all the available pancreatic islet/β-cell models and the possible islet-reactive cytotoxic CD8^+^ T-cell models and *in vitro* microenvironments to understand their crosstalk. Furthermore, the authors discuss the optimum readout parameters for both T-cells and pancreatic β-cells and the prospective of developing such *in vitro* human β-cell killing platforms which will not only help in identifying novel therapeutic targets to halt the T1D progression but also advantageous for the β-cell replacement therapy.

In summary, this Research Topic presents five different papers on understanding and modelling of T1D pathophysiology. It includes two very timely review on the progress of humanized HLA and autoantigen transgenic mice and on human β-cell-T-cell *in vitro* crosstalk platforms for the modelling of human T1D pathogenesis.

## Author contributions

All authors listed have made a substantial, direct, and intellectual contribution to the work and approved it for publication.

